# Inferring predator–prey interactions from camera traps: A Bayesian co‐abundance modeling approach

**DOI:** 10.1002/ece3.9627

**Published:** 2022-12-12

**Authors:** Zachary Amir, Adia Sovie, Matthew Scott Luskin

**Affiliations:** ^1^ School of Biological Sciences University of Queensland St. Lucia Queensland Australia; ^2^ Centre for Biodiversity and Conservation Science University of Queensland St. Lucia Queensland Australia; ^3^ Department of Fisheries and Wildlife Michigan State University East Lansing Michigan USA

**Keywords:** detection probability, hierarchical modeling, N‐mixture models, overdispersion, species interactions, zero inflation

## Abstract

Predator–prey dynamics are a fundamental part of ecology, but directly studying interactions has proven difficult. The proliferation of camera trapping has enabled the collection of large datasets on wildlife, but researchers face hurdles inferring interactions from observational data. Recent advances in hierarchical co‐abundance models infer species interactions while accounting for two species' detection probabilities, shared responses to environmental covariates, and propagate uncertainty throughout the entire modeling process. However, current approaches remain unsuitable for interacting species whose natural densities differ by an order of magnitude and have contrasting detection probabilities, such as predator–prey interactions, which introduce zero inflation and overdispersion in count histories. Here, we developed a Bayesian hierarchical N‐mixture co‐abundance model that is suitable for inferring predator–prey interactions. We accounted for excessive zeros in count histories using an informed zero‐inflated Poisson distribution in the abundance formula and accounted for overdispersion in count histories by including a random effect per sampling unit and sampling occasion in the detection probability formula. We demonstrate that models with these modifications outperform alternative approaches, improve model goodness‐of‐fit, and overcome parameter convergence failures. We highlight its utility using 20 camera trapping datasets from 10 tropical forest landscapes in Southeast Asia and estimate four predator–prey relationships between tigers, clouded leopards, and muntjac and sambar deer. Tigers had a negative effect on muntjac abundance, providing support for top‐down regulation, while clouded leopards had a positive effect on muntjac and sambar deer, likely driven by shared responses to unmodelled covariates like hunting. This Bayesian co‐abundance modeling approach to quantify predator–prey relationships is widely applicable across species, ecosystems, and sampling approaches and may be useful in forecasting cascading impacts following widespread predator declines. Taken together, this approach facilitates a nuanced and mechanistic understanding of food‐web ecology.

## Graphical abstract

Predator–prey dynamics are a fundamental part of ecology, but studying interactions from observational data (e.g., camera trapping) has proven difficult. Here, we developed a Bayesian hierarchical N‐mixture co‐abundance model that is suitable for inferring predator–prey interactions by accounting for excessive zeros and overdispersion in count histories. Our co‐abundance model detected clear positive and negative predator–prey relationships from a large Southeast Asian camera trapping dataset, and we highlight how it overcomes barriers, is widely applicable across species, ecosystems, and sampling approaches, and may be useful in forecasting cascading impacts following widespread predator declines.

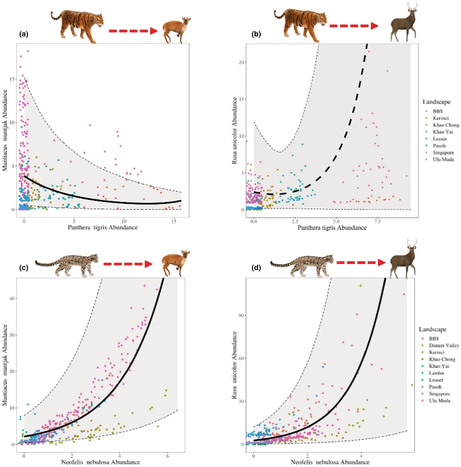

## 1 INTRODUCTION

**FIGURE 1:**
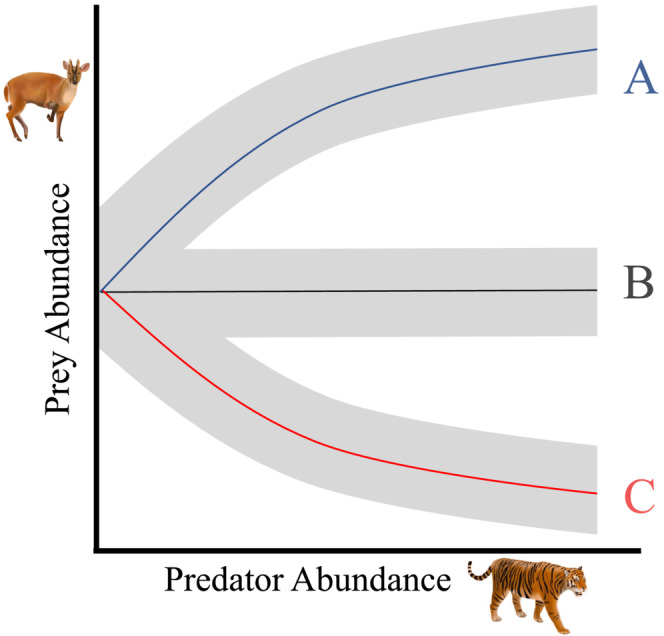
Hypothetical predator–prey co‐abundance relationships. (a) A positive predator–prey relationship suggests top‐down regulation via predation is not the dominant factor shaping abundances. This may arise where there is strong bottom‐up regulation of both prey and predators or severe disturbances affecting both species. (b) The lack of a predator–prey relationship could arise due to a lack of interactions, such as if predator dietary preferences exclude a specific prey species or each species utilizes different habitats. (c) A negative predator–prey relationship could arise from strong top‐down regulation via predation that regulates prey abundance. Similarly, a negative predator–prey relationship suggests predator extirpation may allow prey to increase, termed “trophic release”.

Understanding predator–prey interactions is a foundational theme in ecology (Lotka, 1920; Volterra, 1927). The importance of predator–prey interactions in conservation biology has gained widespread attention following the global decline of apex predators and subsequent trophic cascades (Estes et al., 2011; Ripple et al., 2014). Predators can play keystone roles in structuring ecosystems by regulating prey populations and the spatiotemporal distribution of prey via consumptive and behavioral effects (Estes & Palmisano, 1974; Gaynor et al., 2019). Where predators exert strong top‐down control and suppress prey, predatory interactions can produce a negative co‐abundance relationship (Ripple et al., 2014). In this scenario, predator extirpation may allow prey to increase, termed “trophic release” (Figure 1c; Estes et al., 2011). However, the importance of predators regulating prey across different ecosystems remains debated (Polis & Strong, 1996; Wright et al., 1994). For example, if prey are primarily bottom‐up regulated by limited food availability, and predators are consequentially bottom‐up limited by prey availability, a positive co‐abundance relationship may arise, and this has been observed previously (Figure 1a; Carbone & Gittleman, 2002; Karanth et al., 2004). A positive predator–prey co‐abundance relationship may also suggest alternative forces may be more important than predation in regulating species' abundances (e.g., bottom‐up control, hunting, or other severe disturbances; Ford & Goheen, 2015). Despite the importance of predatory interactions in food‐web ecology and conservation, measuring predator–prey relationships in natural settings imposes several key obstacles due to direct observations, diet analyses, or manipulative experiments being logistically difficult (Smith et al., 2020), especially for cryptic species in tropical forests (Brodie & Giordano, 2013).

Ecologists have frequently used observational methods to study predation by comparing landscapes that vary in predator and prey densities (e.g., Atkins et al., 2019; Brashares et al., 2010; Ripple & Beschta, 2006; Terborgh et al., 2001). However, modern sampling methods that rely on passively observed occurrence data (e.g., from camera traps, acoustic monitors, or eDNA) face numerous analytical barriers to inferring interactions (Blanchet et al., 2020; Ford & Goheen, 2015). Observational studies rarely account for processes influencing both predator and prey, such as environmental and anthropogenic covariates, the complete absence of either species from specific landscapes (i.e., true zeros), and imperfect detection of each species (i.e., false zeros), all of which can bias estimates of interactions (Blanchet et al., 2020; Blasco‐Moreno et al., 2019). For example, generalized joint attribute models can accommodate zero‐inflated and over‐dispersed data to infer species interactions (Clark et al., 2017), but such approaches fail to account for imperfect detection. Recent attempts to use detection‐corrected abundance or co‐occurrence models have failed to propagate uncertainly throughout the modeling process, which may spuriously increase their statistical power (e.g., Penjor et al., 2022). This leaves a gap in our ability to routinely study predator–prey interactions using observational data from standardized biodiversity monitoring programs that now often use camera trapping (Jansen et al., 2014). Here we leverage recent advances in collecting large standardized multispecies datasets via camera trapping and hierarchical N‐mixture co‐abundance models to enhance our understanding of complex wildlife interactions in natural settings.

Many ecological sampling methods produce multispecies detection histories (i.e., binary detections or nondetections per sampling location and sampling occasion) or count histories (i.e., number of individuals counted per sampling location and sampling occasion), including point counts, camera trapping, and acoustic sampling. Detection and count histories can capture the spatiotemporal patterns of species observed across a landscape and have been used to study habitat associations, species distributions, and population dynamics (Kéry & Royle, 2016) but have rarely been used to measure predator–prey relationships (Kéry & Royle, 2021). Detection histories have commonly been used to examine species interactions through several varieties of co‐occurrence models that may quantify nonindependent occurrence (i.e., symmetrical interactions) within the entire community (Tobler et al., 2019) or quantify a directional effect where the occurrence of one species is conditional upon the presence of another (i.e., asymmetrical interactions; Richmond et al., 2010), and both approaches account for imperfect detection. By contrast, count histories have received considerably less attention for inferring species interactions despite carrying more information than detection histories (Roth et al., 2016). Recent work by Blanchet et al. (2020) outlined numerous concerns about using detection histories to infer species interactions and argued that occurrence data does not carry enough information to infer interactions, species occurrence depends on their environment (i.e., habitat filtering), and that strong interactions may lead to exclusions before interactions can be detected.

Co‐abundance models that use count data instead of occurrence data to infer species interactions have solved many of the issues raised by Blanchet et al. (2020; Table 1; Brodie et al., 2018; Roth et al., 2016). Co‐abundance models are an extension of widely used N‐mixture models used to estimate abundance for species that cannot be individually identified (Royle, 2004). N‐mixture models can generate latent population size estimates that are roughly equivalent to capture‐recapture analyses under ideal sampling conditions (Ficetola et al., 2018), though these models can be sensitive to assumption violations that may inflate true population density estimates (Link et al., 2018; Nakashima, 2020). The key advantage of using N‐mixture models is that they accurately quantify the spatial variation in abundance as a function of covariates, thus producing a relative, rather than absolute, a measure of abundance (Gilbert et al., 2021). Currently, co‐abundance models that rely on N‐mixture models to infer species interactions have been limited to competing species that both occur at similar densities so both species' count histories can be assumed to follow a normal Poisson distribution (Brodie et al., 2018; Cosentino et al., 2019; Easter et al., 2020; Roth et al., 2016). Existing co‐abundance models exhibit poor performance and parameter convergence when species count histories have vastly different distributions, limiting their applicability for predators and prey whose natural densities vary by an order of magnitude and exhibit considerably different detection probabilities (Carbone & Gittleman, 2002; Sollmann et al., 2013). Specifically, different population dynamics introduce zero inflation, especially for cryptic species or when species are extirpated from a subset of landscapes, and overdispersion in detections, which is often due to excessive detections at a subset of sampling locations (Blasco‐Moreno et al., 2019; Martin et al., 2005). To move forward studying predator–prey interactions from count histories requires addressing both zero inflation and overdispersion, while simultaneously accounting for imperfect detection and environmental covariates, and incorporating uncertainty throughout the modeling process.

**TABLE 1:** Problems and solutions for inferring species interactions from observational data, such as camera trapping capture histories.

Problems inferring predator–prey interactions	Solutions
Occurrence data (i.e., presence and absence) lacks enough information to infer biotic interactions (Blanchet et al., 2020; Roth et al., 2016)	Camera trapping can obtain count data (i.e., number of individuals per independent capture) and N‐mixture models utilize count data in a count history matrix to estimate abundance while accounting for imperfect detection (Royle, 2004)
Need to propagate uncertainty throughout the entire modeling process (Brodie & Giordano, 2013)	Use integrated co‐abundance model where all parameters are estimated in a single set of simulations using Bayesian Markov chain Mote Carlo (MCMC) methods (Brodie et al., 2018; Roth et al., 2016)
Species occurrence and abundance depend on the environment (i.e., habitat filtering) and the joint habitat preferences of two species could falsely create the illusion of interactions (Blanchet et al., 2020; Dormann et al., 2018)	Integrate environmental covariates affecting both species into the co‐abundance modeling framework (Brodie et al., 2018; Roth et al., 2016)
Sampling scale influences measures of co‐occurrence. Spatial scale must be fine scale enough to detect interactions between individuals, while also encompassing broad variation in species' distributions (Blanchet et al., 2020)	Spatially resample camera trap locations to reflect the study species' home ranges that ensure detections are spatially independent and comparable across camera trapping sessions (Rayan & Linkie, 2020). Also, use a large multi‐landscape dataset that represents diverse samples of both species' distributions
Appropriate statistical inference requires a very large sample size (Blanchet et al., 2020)	Technological and cost improvements have made it possible to conduct large, standardized, and repeated camera trapping sessions that can collectively generate sufficient sample sizes, even for rare and cryptic species. For example, in our case study below, we used data from 1210 camera traps from 20 sessions conducted across 10 landscapes that generated 5980 independent captures of our four study species
Strong interactions may lead to complete species exclusions and adding zeros to count histories usually leads to deviations from the normal Poisson distribution (Blanchet et al., 2020)	Incorporate species absences in count histories and classify zeros as true or false zeros (Blasco‐Moreno et al., 2019). Account for true zeros (e.g., species extirpated from a landscape) using an informed zero‐inflated Poisson distribution, and account for false zeros (e.g., present but not detected) by correcting for imperfect detection
Positive correlations between predator and prey abundances are difficult to interpret in terms of interspecific interactions (Brodie et al., 2018)	Interpret negative predator–prey relationships as evidence that predation is regulating the focal prey population(s) (Ripple et al., 2014). Interpret positive predator–prey relationships as evidence that predation is not the primary force regulating the focal prey population(s). Positive predator–prey relationships suggest alternative forces (e.g., bottom‐up control, hunting) may be more important in shaping the focal prey species abundance (Ford & Goheen, 2015)
N‐mixture models are sensitive to model assumption violations that can inflate absolute density estimates leading to inaccurate inferences for determining population size (Link et al., 2018)	Interpret results from co‐abundance models as the directional change in species abundance relative to covariates and not as absolute population sizes (Gilbert et al., 2021)

*Note*: This manuscript describes an approach to implement all solutions.

We developed a Bayesian hierarchical N‐mixture co‐abundance model to study predator–prey interactions while conforming to the criteria proposed by Blanchet et al. (2020) for robust inferences (Table 1). Our key improvements over existing co‐abundance models for competition (Brodie et al., 2018; Cosentino et al., 2019; Easter et al., 2020) are introducing an informed zero‐inflated Poisson distribution in the abundance formula and accounting for overdispersion in detections using a random effect per sampling unit and sampling occasion in the detection formula. We illustrate that the combination of these two methodological advancements is necessary to infer ecologically meaningful predator–prey interactions while ensuring parameter convergence and goodness‐of‐fit across several different species pairs. Our co‐abundance models facilitate the study of predator–prey interactions across trophic levels by quantifying predator–prey co‐abundance relationships (Figure 1), while accounting for each species' detection probability, relationships with environmental covariates, and propagates uncertainty. As a real example, we examined four potential predatory interactions using a multi‐session multi‐landscape camera trapping dataset from Southeast Asian tropical forests. We quantified predator–prey co‐abundance relationships to test whether tigers (*Panthera tigris*) or clouded leopards (*Neofelis nebulosa* and *N. diardi*) suppress sambar deer (*Rusa unicolor*) or muntjac deer (*Muntiacus muntjac*).

## 2 MATERIALS AND METHODS

### 2.1 A two‐species N‐mixture model

Our approach was to test for the effect of a dominant predator's local abundance on a subordinate prey's local abundance using count histories. We adapted the N‐mixture co‐abundance modeling framework from Brodie et al. (2018) that included a term for the latent abundance of a dominant species affecting the abundance of a subordinate species. This N‐mixture model estimates local abundance for species *i* (either dominant _dom_ or subordinate _sub_) at sampling unit *j*, denoted as *N*_*i,j*_, through repeated counts of the population over a time frame during which the population is closed to change (Royle, 2004). We assume that:

$$N_{i,j}∼\text{Poisson}\;\left(\lambda_{i,j}\right)$$

and model the expected count of species *i* at sampling unit *j*, λ_*i,j*_, relative to covariates using a log‐link function (Royle, 2004). However, we expanded upon Brodie et al.'s approach by including informed zero‐inflated Poisson (hereafter “iZIP”) distributions for both dominant and subordinate species to account for true zeros in count history matrices when either species was known to be absent. Traditional uniformed ZIP N‐mixture models define the occupancy status of species *i* at sampling unit *j*, Z_*i,j*_ as a random Bernoulli trial and multiply the expected count of species *i* at sampling unit *j*, *λ*_*i,j*_, by *Z*_*i,j*_ (Blasco‐Moreno et al., 2019; Kéry & Royle, 2016; Martin et al., 2005). We informed the occupancy status of species *i* at sampling unit *j*, *Z*_*i,j*_ based on our observational camera trapping data where *Z*_*ij*_ was 1 if the sampling unit was in a landscape where the species was detected, and *Z*_*ij*_ was 0 in the event the species was never detected and existing literature corroborated their extirpation (Amir et al., 2022; Blasco‐Moreno et al., 2019; Martin et al., 2005). Therefore, our iZIP N‐mixture model assumes that:

$$N_{i,j}∼\text{Poisson}\;\left({\lambda_{i,j}}^{*}\;Z_{\mathrm{ij}}\right)$$

Fixing local abundance to zero rather than estimating nonzero abundance where the species was extirpated minimizes the chances of making a type I error (Martin et al., 2005).

The effect of the covariates and the dominant on the subordinate species was modeled as:

$$\log\left(\lambda_{\mathrm{sub},j}\right)={\alpha_{\mathrm{sub}}}^{*}\;{\text{covariates}}_{j}+\delta^{*}\;N_{\mathrm{dom},j}$$

where α_sub_ is a vector of environmental covariate effects for the subordinate and *δ* represents the effect of the latent abundance per sampling unit of the dominant species (*N*_dom,*j*_) on the subordinate. An estimated value of *δ* < 0 would infer a negative co‐abundance relationship between the dominant and subordinate species in support of a predatory interaction (e.g., top‐down regulation; Figure 1c). An estimated value of *δ* > 0 would infer a positive predator–prey co‐abundance relationship that is likely driven by responses to unmodelled covariates (e.g., food availability to prey that underlies bottom‐up regulation, Figure 1a). Estimates of *δ* with 95% Bayesian credible intervals (CI) overlapping zero do not indicate biologically meaningful interactions (Figure 1b). Assuming the covariates included in this model are appropriate, this approach allows us to tease apart the directional impact of a dominant on subordinate species (Brodie et al., 2018). The abundance model for both dominant and subordinate species included the iZIP parameter and the same environmental covariates, while the subordinates included the additional parameter estimating the effect of the latent abundance of the dominant species *δ* * *N*_dom,*j*_.

We assume that we imperfectly observed the latent abundance of both species during sampling, thus giving rise to false zeros in our count histories (Blasco‐Moreno et al., 2019; Royle, 2004). Abundance cannot be directly observed at sampling units, so sampling biases like imperfect detection are accommodated in estimates of *N*_*i,j*_ by assuming that the detections of species *i* at sampling unit *j* during sampling occasion *k*, denoted as *n*_*i,j,k*_, follow a binomial distribution with species‐level detection probability *p*_*i,j,k*_:

$$n_{i,j,k}∼\text{Binomial}\;\left(N_{i,j,k},p_{i,j,k}\right)$$

We modeled the detection probability of species *i* at sampling unit *j* during sampling occasion *k*, *p*_*i,j,k*_, relative to covariates using a logit‐link function (Royle, 2004), and we used the same sampling‐related covariates in the detection model for both species. The original N‐mixture model with a binomial detection process assumes that individuals are not double‐counted between sampling occasions and sampling units (Royle, 2004), and this assumption may be violated if the same individual is detected multiple times within the same sampling occasion. Double counting individuals can lead to inflated abundance estimates, so there have been suggestions to address this using a Poisson detection process, which we also tested (Link et al., 2018; Nakashima, 2020). To account for overdispersion in species‐specific detection probability not captured by sampling‐related covariates, we included a random effect per sampling unit and sampling occasion (an overdispersion random effect, hereafter “ODRE”) per species, ε_*i,j,k*_ (Kéry & Royle, 2016; Roth et al., 2016). For the ODRE, we assume that:

$$\varepsilon_{i,j,k}∼\text{Normal}\left(0,\tau_{i}\right)$$

where *τ*_*i*_ was the standard deviation of the ODRE. We also included a stabilizing parameter to ensure the logit‐link transformation would not become zero or negative (Kéry & Royle, 2016). Bringing the sampling‐related covariates and ODRE together, we define the detection probability of species *i* at sampling unit *j* on sampling occasion *k* as:

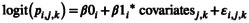

We estimated model parameters using a Bayesian approach with MCMC methods with the program R (R Development Core Team, 2021) in the package “jagsUI” (Kellner, 2019). We used this Bayesian approach to propagate uncertainty throughout the modeling process by first estimating the latent abundance of the dominant species per sampling unit, *N*_dom,*j*_, which is then used as a covariate to estimate the latent abundance of the subordinate per sampling unit, *N*_sub,*j*_ (Brodie et al., 2018; Roth et al., 2016). As our primary goal for this N‐mixture co‐abundance model was the examine the directional impact of a dominant species' local abundance upon a subordinate species' local abundance, and not to estimate latent population sizes, we refrain from interpreting our results in terms of absolute density but rather as the spatial variation in abundance relative to covariates (i.e., *δ* * *N*_dom*j,k*_; Gilbert et al., 2021). Apart from our iZIP parameter (*Z*_*ij*_), we used uninformative prior values and provided similar initial values close to zero for all parameters. For each species pair, we ran three chains of 1,000,000 iterations because Kéry and Royle (2016) stressed the importance of running long chains when incorporating the ODRE. We discarded the first 200,000 iterations as burn‐in and thinned the chains by 80, which left 30,000 values to quantify the posterior distribution of each parameter. We assumed parameters converged if their Rhat scores were between 1 and 1.2 (Gelman et al., 2013). We calculated the 95% CI from the posterior distribution and considered our parameters to have a clear effect (or “significant effect” in frequentist terminology) if 0 was not included in the 95% CI (Roth et al., 2016). To infer confidence about parameter directionality, we calculated the probability the posterior distribution of the species interaction parameter was either negative or positive using the R package “bayestestR” (Makowski et al., 2019). Finally, our model has the same assumptions as a standard N‐mixture model, including independence among sampling units, population closure over all replicated sampling occasions, independent and equal detection probabilities among individuals within a species, and that abundance per sampling unit was a random variable with *E*(*N*_*j*_) = λ (Royle, 2004).

### 2.2 Evaluating model performance

We assessed model performance by inspecting model goodness‐of‐fit and the magnitude of overdispersion by calculating “Bayesian *p*‐values” and “C‐hat” scores via χ^2^ discrepancies using posterior predictive checks (PPC) (Conn et al., 2018; Gelman et al., 1996). The PPC simulates a count history matrix from the joint posterior distribution and estimates the level of consistency with the observed count history matrix. Bayesian *p*‐values reflect the proportion of times the simulated data were greater than the observed data, and C‐hat values reflect the magnitude of overdispersion by dividing the observed data by the simulated data. Bayesian *p*‐values between .25 and .75 indicate a good fit, values that equal 0.5 indicate a perfect fit, and values outside this range indicate a lack of fit (Conn et al., 2018; Gelman et al., 1996; Kéry & Royle, 2016) and C‐hat values greater than 1.1 suggest remaining overdispersion (Kéry & Royle, 2016; Mazerolle, 2020). Finally, we assessed Bayesian *p*‐values, C‐hat scores, and the species interaction parameter using Rhat scores and the posterior estimates to compare model performance across four model structures: (i) the original Poisson model of Brodie et al. (2018), (ii) the iZIP model, (iii) the Poisson + ODRE model, and (iv) the final models that include the iZIP + ODRE.

### 2.3 Example: Southeast Asian predator–prey dynamics

#### 2.3.1 Camera trapping methods

We assessed predator–prey co‐abundance relationships using a large multi‐session multi‐landscape camera trap dataset from Southeast Asian tropical forests. We conducted 20 camera trapping sessions in ten lowland primary tropical forest landscapes in Sumatra (3), Borneo (2), Singapore (1), Peninsular Malaysia (2), and Thailand (2) (Table S1). We refer to sampling areas as a “landscape” and these all include protected areas as well as some nearby production forests and forest patches. For detailed landscape descriptions, see Amir et al. (2022). At each landscape, we deployed 22–112 passive infrared camera traps set across areas of 48–830 km^2^. We standardized camera deployment between landscapes using either Reconyx or Bushnell cameras attached to trees at 0.2–0.3 m height and placed along natural wildlife trails without baits (Jansen et al., 2014). We deployed cameras for approximately 30–90 days (mean = 40.4, SD = 31.6) to ensure population closure assumptions.

We systematically deployed camera traps in each landscape in a grid format and then spatially resampled the camera traps into 7.8 km^2^ hexagonal sampling units due to unequal camera spacing between large continuous forests (>1 km between cameras) and small forest fragments (<500 m between cameras). Resampling all camera traps into spatially standardized sampling units ensures comparability among landscapes, prevents spatial pseudo‐replication, and ensures that we are estimating abundance as opposed to habitat use (Rayan & Linkie, 2020). When multiple cameras occurred in a single sampling unit, we averaged their covariate values and aggregated the number of individuals observed per day. We considered captures of the same species independent if they occurred at least 30 min apart (Rovero & Zimmermann, 2016). We grouped our count data into 5‐day sampling occasions to decrease the number of false zeros in the dataset (i.e., sampling occasions with no detections) and increase detection probabilities (Brodie et al., 2018).

#### 2.3.2 Study species

Tigers (*Panthera tigris*) are the largest carnivores distributed across Southeast Asia, and clouded leopards (*Neofelis nebulosa* and *N. diardi*, analyzed as one species) are large carnivores that co‐occur with tigers across mainland Southeast Asia and Sumatra but are the largest carnivores in Borneo where tigers are no longer native (Phillipps & Phillipps, 2016). We only included count data from landscapes where the predator species is native, which allowed us to incorporate landscapes where the species is extirpated (e.g., tigers in Singapore) and exclude landscapes where the species is not native (e.g., tigers in Borneo, Amir et al., 2022). Sambar deer (*Rusa unicolor*) and muntjac deer (*Muntiacus muntjak*) deer are common large ungulates widely distributed through our study region. For gregarious muntjac deer (20+ individuals per sampling occasion), we followed Brodie et al. (2018) by analyzing the number of muntjac groups rather than the number of individuals in the count history and limited the daily observations to zero or one group. A systematic range‐wide dietary study suggests tigers preferentially prey upon the largest available prey species with a weight range from 60 to 250 kg, such as sambar deer (Hayward et al., 2012), while single‐landscape studies highlight muntjac deer as an important prey species for clouded leopards (Can et al., 2020; Petersen et al., 2020). Therefore, we expected strong negative co‐abundance relationships between tigers and sambar deer and between clouded leopards and muntjac deer in support of predatory interactions.

#### 2.3.3 Covariates

While there are many factors that affect species abundance, our analysis focused on a limited set of powerful composite variables. To account for environmental and anthropogenic disturbances that may affect species abundance, we included the Forest Landscape Integrity Index (FLII) and Human Footprint Index (HFP) as fixed effect covariates on the abundance formula for both species. The FLII is a globally continuous measure of the world's forests that integrates both observed deforestation and inferred edge effects and the loss of connectivity (Grantham et al., 2020). The HFP is a globally continuous measure that represents landscape‐level anthropogenic disturbances from human population densities and infrastructure, and can be used as a crude metric to infer potential hunting pressure (Venter et al., 2016). We calculated FLII and HFP values in QGIS for every camera trap location. We also included a random‐intercept effect in our abundance formula to account for unmodeled variation between landscapes and to account for three landscapes with repeated sampling. Finally, we included a fixed effect for sampling effort (in trap nights) per sampling unit in our detection probability formula to account for multiple cameras being resampled into the same sampling unit and unequal deployment lengths. We standardized all numeric covariates (mean = 0, SD = 1) and examined Pearson correlation coefficients, and ensured no collinearity among covariates (|*r*| < 0.5). The abundance formula modeled the expected count relative to environmental covariates for species *i* at sampling unit *j* as follows:

$$\log\left(\lambda_{i,j}\right)={\text{α0}}_{i}+{{\text{α1}}_{i}}^{*}\;{\text{FLII}}_{j}+{{\text{α2}}_{i}}^{*}\;{\mathrm{HFP}}_{j}+{{\text{α3}}_{i}}^{*}\;{\text{Landscape}}_{j}$$

The abundance model for the subordinate included an additional parameter estimating the effect of the latent abundance of the dominant species: *δ* * *N*_dom,*j*_. Our detection probability formula was the same for both predator and prey and modeled the detection probability of species *i* at sampling unit *j* on sampling occasion *k* as:

$$\text{logit}\left(p_{i,j,k}\right)={\text{β0}}_{i}+{{\text{β1}}_{i}}^{*}\;{\text{Effort}}_{j}+\varepsilon_{i,j,k}$$

## 3 RESULTS

### 3.1 Model performance

**FIGURE 2:**
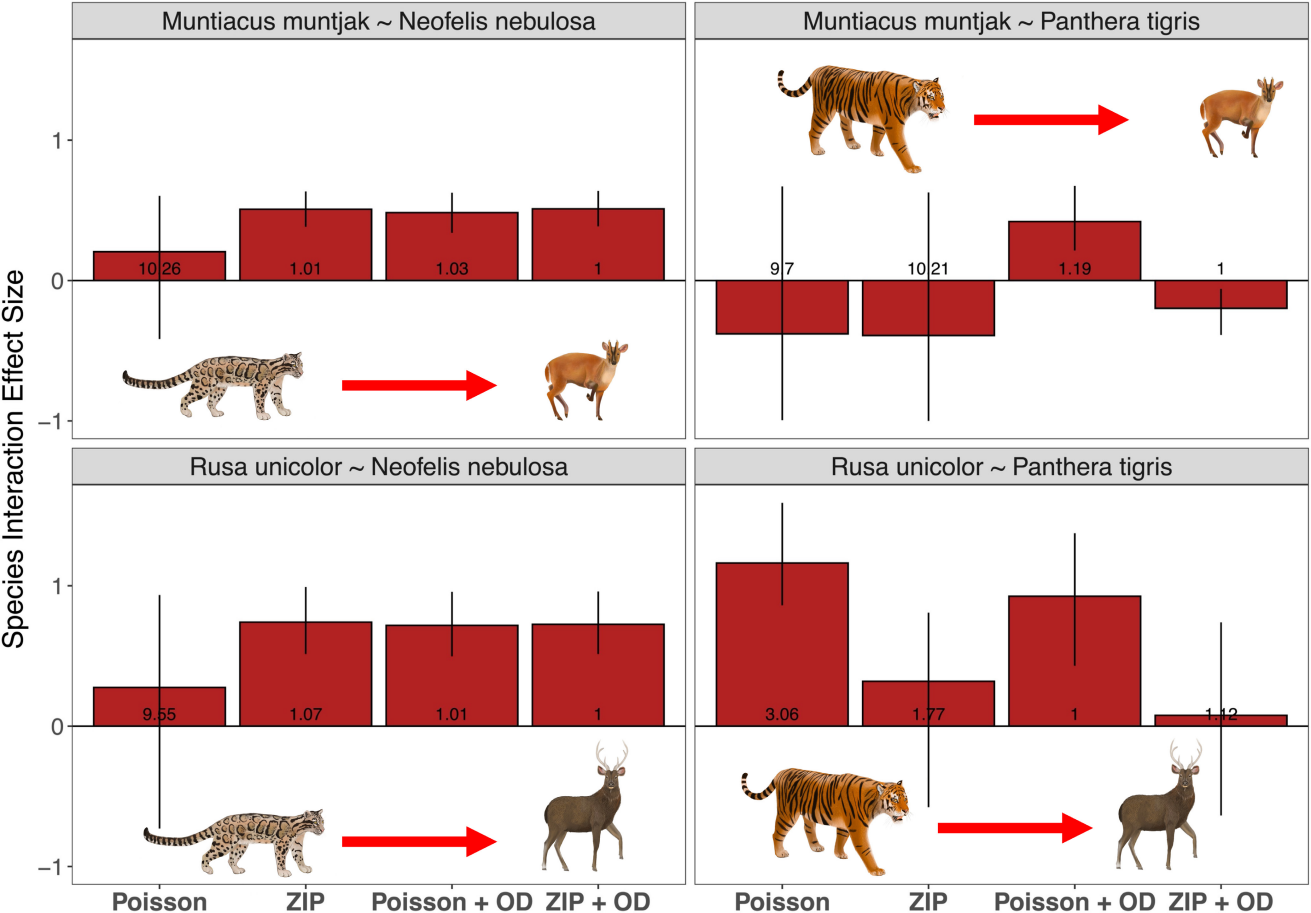
Comparing the effect sizes, 95% Bayesian credibility interval, and parameter convergence (Rhat values) of the species interaction parameter in all four species pairs from our two‐species N‐mixture models. We examined the original two‐species N‐mixture model proposed by Brodie et al. (2018) (*Poisson*), then examined the addition of the iZIP parameter (*ZIP*) and ODRE parameter (*Poisson + OD*) separately, and finally compared our final models that include both the iZIP and ODRE parameters (*ZIP + OD*). The Y‐axis represents the mean effect sizes of the species interaction parameter, and the error bars represent the 95% Bayesian credibility interval. Finally, the values at the bottom of the bar graphs represent the specific Rhat scores from the species interaction parameter associated with each model.

**FIGURE 3:**
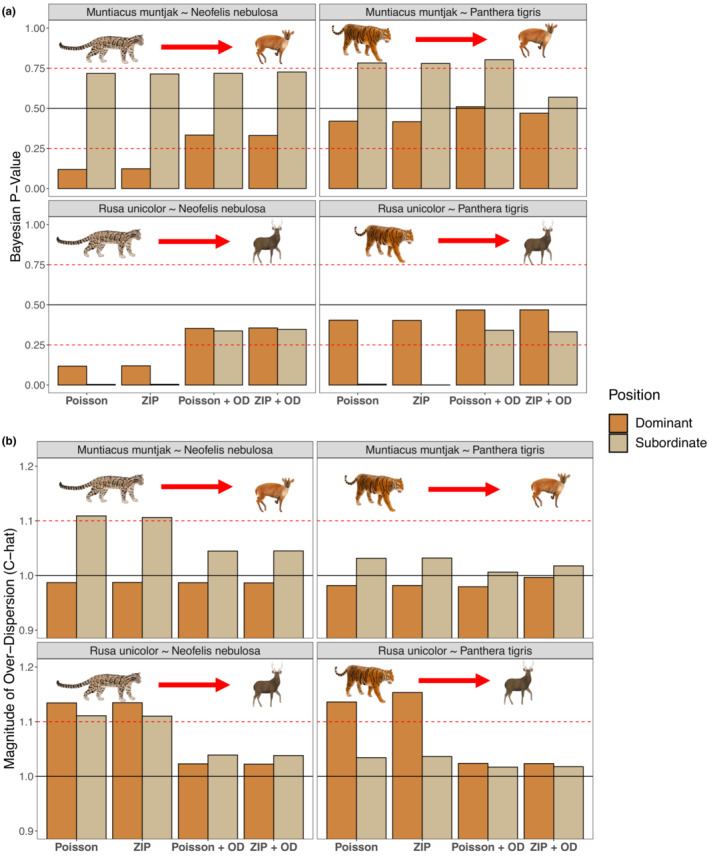
Comparing the goodness‐of‐fit between models by inspecting Bayesian *p*‐values (a) and the magnitude of overdispersion C‐hat values (b) across four species pairs. We examined the original two‐species N‐mixture model proposed by Brodie et al. (2018) (*Poisson*), then examined the addition of the iZIP parameter (*ZIP*) and ODRE parameter (*Poisson + OD*) separately, and finally compared our final models that include both the iZIP and ODRE parameters (*ZIP + OD*). (a) Bayesian *p*‐values are calculated by taking the mean value of the number of times data simulated from the joint posterior distribution was greater than the real data supplied to the model, where Bayesian *p*‐values between .25 and .75 indicate good fit, a value of 0.5 indicates a perfect fit, and values above or below the dashed red lines (<0.25 or >0.75) indicate a lack of fit. (b) C‐hat values are calculated by dividing the observed data supplied to the model from data simulated from the joint posterior distribution and we visualized the mean value, where C‐hat values greater than 1.1 indicate remaining overdispersion and values close to 1 indicate no remaining overdispersion. A horizontal line is added at 1 to indicate the ideal value for our C‐hat scores, while the red dashed line at 1.1 denotes our cut‐off point for C‐hat values that suggest overdispersion. Both dominant (darker tan color) and subordinate (lighter tan color) species are included in both figures.

Our co‐abundance modeling approach that accounted for zero inflation and overdispersion in count histories using both the iZIP distribution and ODRE successfully converged across all species pairs (Rhat <1.2; Table 2; Figure 2). Including either the iZIP and ODRE parameters improved model goodness‐of‐fit (Bayesian *p*‐values were closer to .5 than models lacking these parameters, Figure 3a) and reduced overdispersion (C‐hat values were between 1.1 and 1.0; Figure 3b) compared with models that lacked one or both improvements. Including both the iZIP and ODRE parameters improved model performance the most, as illustrated by the highest convergence around the species interaction parameter, Bayesian *p*‐values closest to .5, and C‐hat values that suggest no remaining overdispersion. The key exception was for the model examining the impact of tigers on sambar deer, which showed improved convergence and a clear species interaction effect when omitting the iZIP distribution and including the ODRE (Table 2b). Co‐abundance models using a Poisson instead of a binomial detection process produced lower absolute population size estimates for most species but followed the same relative abundance trends between landscapes (Figure S1) and equivalent directionality for our species interaction parameter (*δ* * *N*_dom,*j*_; Figure S2). However, using a Poisson detection formula models exhibited unacceptable goodness‐of‐fit (Bayesian *p‐*values >.75) for two‐species pairs (Figure S3) and reduced precision of the species interaction parameter that undermined its biological and applicable utility. Therefore, we present co‐abundance models using the binomial detection formula in the main text and the Poisson detection formula in the supplement.

**TABLE 2:** Summary of key results from comparing four progressively developed versions of the co‐abundance models across four species pairs.

(A) Predator–prey interaction: muntjac deer ~ tigers 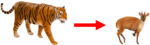						
Model	Species interaction	Rhat	Muntjac deer Bayes *p*‐value	Muntjac deer C‐hat	Tiger Bayes *p*‐value	Tiger C‐hat
Poisson	−0.38 (±0.61)	9.70	.78	1.03	.42	0.98
iZIP	−0.39 (±0.60)	10.2	.78	1.03	.42	0.98
Poisson + OD	0.42 (±0.12)	1.18	.80	1.01	.51	0.98
iZIP + OD	−0.20 (±0.09)	1.00	.57	1.02	.47	1.00

(B) Predator–Prey interaction: sambar deer ~ tigers 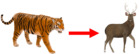						
Model	Species interaction	Rhat	Sambar deer Bayes *p*‐value	Sambar deer C‐hat	Tiger Bayes *p*‐value	Tiger C‐hat
Poisson	1.16 (±0.21)	3.06	.006	1.03	.40	1.14
iZIP	0.32 (±0.39)	1.77	.0008	1.04	.40	1.15
Poisson + OD	0.93 (±0.23)	1.00	.34	1.02	.47	1.02
iZIP + OD	0.07 (±0.45)	1.12	.33	1.02	.47	1.02

(C) Predator–Prey interaction: muntjac deer ~ clouded leopards 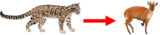						
Model	Species Interaction	Rhat	Muntjac deer Bayes *p*‐value	Muntjac deer C‐hat	Clouded leopard Bayes *p*‐value	Clouded leopard C‐hat
Poisson	0.21 (±0.39)	10.3	.72	1.11	.12	0.99
iZIP	0.51 (±0.06)	1.01	.71	1.11	.12	0.99
Poisson + OD	0.48 (±0.07)	1.03	.72	1.04	.33	0.99
iZIP + OD	0.51 (±0.07)	1.00	.73	1.04	.33	0.99

(D) Predator–Prey interaction: sambar deer ~ clouded leopards 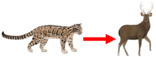						
Model	Species Interaction	Rhat	Sambar deer Bayes *p*‐value	Sambar deer C‐hat	Clouded leopard Bayes *p*‐value	Clouded leopard C‐hat
Poisson	0.26 (±0.61)	9.55	.004	1.11	.12	1.13
iZIP	0.74 (±0.12)	1.07	.005	1.11	.12	1.13
Poisson + OD	0.72 (±0.12)	1.01	.34	1.04	.35	1.02
iZIP + OD	0.73 (±0.11)	1.00	.35	1.04	.36	1.02

*Note*: We started with the original two‐species N‐mixture model developed by Brodie et al. (2018) (*Poisson*). We compared this to models using either the informed zero‐inflated Poisson distribution (*iZIP*), or a random effect per sampling unit and sampling occasion (*OD*), and to the models presented in the main text using both (*iZIP + OD*). The species interaction column shows the posterior distribution's mean and standard deviation of the species interaction parameter, and Rhat values between 1 and 1.2 denote that the parameter successfully converged. Bayesian *p*‐values between .25 and .75 indicate a suitable goodness‐of‐fit, a value of .5 indicates a perfect fit, and values outside this range indicate unacceptable performance. C‐hat values greater than 1.1 indicate unacceptable overdispersion.

### 3.2 Southeast Asian predator–prey dynamics

**FIGURE 4:**
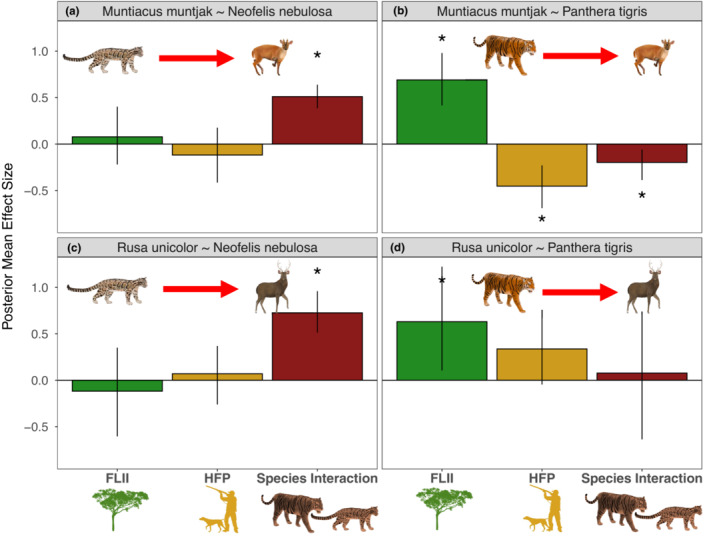
Parameters describing prey species abundance from the Bayesian co‐abundance models using the informed zero‐inflated Poisson (iZIP) distribution and ODRE. Plots show the posterior mean effect size, and the error bars represent the 95% Bayesian credibility interval (CI), with asterisks (*) denoting relationships where the 95% CI does not include zero. The variables are FLII (green), HFP (yellow), and the species interaction (red), which shows the effect of the dominant (predator) on the subordinate (prey).

**FIGURE 5:**
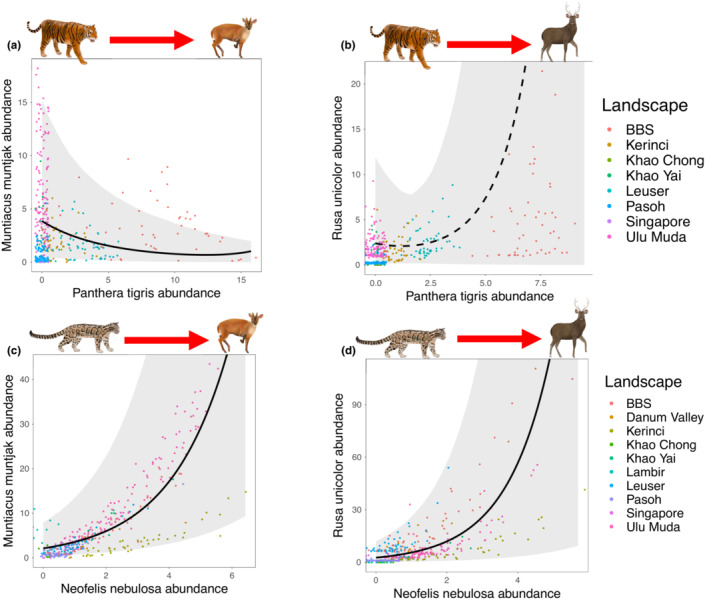
Predator–prey relationships estimated from the Bayesian co‐abundance models using the iZIP distribution and ODRE. The thick black trend line comes from the posterior distribution of the species interaction parameter, the gray shaded area shows the 95% Bayesian credibility interval (CI), and a solid trend line indicates the 95% CI does not include zero. The points show the estimated species abundances at each sampling unit, colored by landscape. The landscape abbreviations used in the legend are as follows: BBS refers to Bukit Barisan Selatan National Park, Danum Valley refers to Danum Valley Conservation Area, Kerinci refers to Kerinci Seblat National Park, Khao Chong refers to Khao Ban Tat Wildlife Sanctuary, Khao Yai refers to Khao Yai National Park, Lambir refers to Lambir Hills National Park, Leuser refers to Gunung Leuser National Park, Pasoh refers to Pasoh Forest Reserve, Singapore refers to the Central Catchment Nature Reserve and Palau Ubin, and finally Ulu Muda refers to the Greater Ulu Muda Forest Complex.

We collected 5980 independent captures of our four study species over a sampling effort of 58,071 trap nights (Tables S1 and S2). We observed both clear (i.e., the 95% CI does not include 0) negative and positive predator–prey relationships from our co‐abundance models (Figures 4 and 5). The detection probability of all species assessed across all species pairs showed a clear positive relationship with sampling effort (>99% probability; Figure S4).

### 3.3 Muntjac ~ tiger

The abundance of tigers clearly and negatively influenced muntjac deer abundance [Posterior mean effect size (hereafter “ES”) = −0.20, 95% CI = −0.39 to −0.06, >99% probability that posterior distribution is negative; Figures 4a and 5a]. Tiger abundance showed a clear positive relationship with FLII (ES = 0.82, 95% CI = 0.31–1.46, >99% probability) and a weak negative relationship with HFP (ES = −0.10, 95% CI = −0.49 to 0.22, 72% probability). Muntjac deer abundance showed a clear positive relationship with FLII (ES = 0.69, 95% CI = 0.42–0.98, >99% probability) and a clear negative relationship with HFP (ES = −0.45, 95% CI = −0.69 to −0.23, >99% probability).

### 3.4 Sambar ~ tiger

There was no clear relationship between tigers and sambar deer (ES = 0.08, 95% CI = −0.64 to 0.74, 57% probability; Figures 4b and 5b). Tiger abundance did not show clear associations with FLII (ES = 0.17, 95% CI = −0.35 to 0.89, 65% probability) of HFP (ES = −0.12, 95% CI = −0.61 to 0.32, 63% probability). Sambar deer abundance showed a clear positive association with FLII (ES = 0.63, 95% CI = 0.11–1.2, >99% probability) and a positive association with HFP (ES = 0.34, 95% CI = −0.05 to 0.76, 94% probability).

### 3.5 Muntjac ~ clouded leopard

Muntjac deer were clearly and positively associated with clouded leopards (ES = 0.51, 95% CI = 0.39–0.64, >99% probability; Figures 4c and 5c). Clouded leopard abundance showed a clear positive association with FLII (ES = 0.37, 95% CI = 0.01–0.80, 98% probability) and a weak negative association with HFP (ES = −0.10, 95% CI = −0.39 to 0.18, 76% probability). Muntjac deer abundance did not show a clear relationship with FLII (ES = 0.08, 95% CI = −0.22 to 0.40, 68% probability) or HFP (ES = −0.12, 95% CI = −0.42 to 0.18, 79% probability).

### 3.6 Sambar ~ clouded leopard

Sambar deer were clearly and positively associated with clouded leopards (ES = 0.73, 95% CI = 0.51–0.96, >99% probability; Figures 4d and 5d). As with the muntjac deer model, clouded leopard abundance showed a clear positive relationship with FLII (ES = 0.68, 95% CI = 0.23–1.28, >99% probability) and a weak positive association with HFP (ES = 0.12, 95% CI = −0.16 to 0.39, 82% probability). Sambar deer abundance did not show clear relationships with FLII (ES = −0.12, 95% CI = −0.6 to 0.35, 69% probability) or HFP (ES = −0.07, 95% CI = −0.26 to 0.37, 69% probability).

## 4 DISCUSSION

We introduced a two‐species N‐mixture modeling approach to quantify predator–prey relationships from observational count histories, while accounting for shared responses to the environment and propagating uncertainty throughout the modeling process. We assessed how this model performed using camera trapping data of two Asian apex predators and two of their key prey species that exhibit contrasting detection probabilities and natural densities. We found that explicitly classifying both the source of true zeros with the iZIP distribution in the abundance formula and false zeros with our detection formula containing the ODRE were necessary to infer ecologically meaningful predator–prey interactions while ensuring parameter convergence across all species pairs (Blasco‐Moreno et al., 2019; Martin et al., 2005). Failing to classify true zeros, such as at landscapes where a species has been extirpated, leaves the model to estimate nonzero abundance due to imperfect detection (i.e., false zeros) that leads to overconfidence in the posterior estimates and increases the risk of a type I error (Martin et al., 2005). We illustrated this type I error by showing a spurious clear positive relationship between tigers and sambar deer when excluding the iZIP parameter, which disappeared when using the iZIP distribution. Our approach also produced relationships with environmental covariates across all species that are supported by past research suggesting anthropogenic pressure suppresses ungulate abundances and that intact tropical forests support large carnivore abundances (Hearn et al., 2019; Luskin, Albert, & Tobler, 2017; Macdonald et al., 2019; Rayan & Linkie, 2020).

There is a strong appetite to infer species interactions from camera trap data and to overcome the limitations of traditional co‐occurrence models (Blanchet et al., 2020). While our approach is fit‐for‐purpose, there are several drawbacks including these models being data hungry, computationally demanding, and statistically complicated. To overcome data limitations, such as for tigers in our datasets that were only detected only in three of ten sampled landscapes, collaborative projects may be required to achieve sufficient sample sizes and span a wide gradient of predator abundances. Another solution may be to integrate multiple data sources such as direct observations with camera trapping (Miller et al., 2019). The computational power required to run and test several models with 1,000,000 MCMC iterations may pose a considerable barrier for many field ecologists (Pettorelli et al., 2021). Access to high‐performance research computing that simultaneously solves multiple models while distributing MCMC chains across multiple cores will save researchers a substantial amount of time in such analyses (Visser et al., 2015). Finally, there are concerns about the reliability of N‐mixture models when assumptions are violated, such as when the same individual is observed multiple times in a single sampling occasion (Link et al., 2018). This violation can cause inflation in absolute population size estimates, so if researchers require accurate latent population size estimates, we encourage the use of a Poisson detection process (Nakashima, 2020). We implemented co‐abundance models with both binomial and Poisson detection processes and observed equivalent directionality and similar species interaction parameters, suggesting either detection process may be suitable for examining the spatial variation in abundance (Gilbert et al., 2021).

Predator losses in temperate ecosystems often lead to trophic release, where a subset of prey species increase in abundance (Ripple et al., 2009), but predator–prey relationships appear weak or positive in tropical forests (Brodie & Giordano, 2013). Predator–prey relationships in tropical forests may be strongly shaped by diffuse food webs with functionally redundant links, strong bottom‐up control, and/or the joint suppression of many animals by overwhelming disturbances such as defaunation (Benítez‐López et al., 2019; Brodie & Giordano, 2013; Polis & Strong, 1996; Wright et al., 1994). In accordance with this work, we found only one clear negative relationship suggesting tigers regulate muntjac deer. Further, we detected positive predator–prey relationships between clouded leopards and both muntjac and sambar deer, suggesting predation is not the dominant force regulating Asia's deer. Instead, prey abundance may be a fundamental determinant of predator abundance (Carbone & Gittleman, 2002; Karanth et al., 2004) with deer being noted as key prey for clouded leopards (Can et al., 2020; Petersen et al., 2020). These results also suggest that hunting deer may suppress clouded leopards via prey depletion (Wolf & Ripple, 2016), or hunting may jointly suppress clouded leopards and deer together (Benítez‐López et al., 2019; Ford & Goheen, 2015). Future research could apply our modeling approach to reverse the role of dominant and subordinate species to examine whether the abundance of clouded leopards is positively associated with deer abundance, thereby testing support for bottom‐up regulation.

This modeling framework is widely applicable across species, ecosystems, and sampling approaches (acoustic monitoring, camera trapping, and point counts) that produce count histories. A key improvement of our co‐abundance modeling approach that classifies the source of zeros in count histories is to move trophic cascades research past binary comparisons of landscapes based on predator presence or absence (e.g., Atkins et al., 2019; Brashares et al., 2010) to a gradient of predator abundance, including where predators are absent. Our modeling framework incorporates covariates and has the potential to explain divergent co‐abundance patterns across environmental gradients, such as edge‐adapted and disturbance‐tolerant species proliferating in predator‐free forest fragments (Terborgh et al., 2001). Future research could use this co‐abundance model to examine how predator–prey relationships vary across ecological gradients, which has important conservation implications for predicting trophic cascades (Terborgh, 2015).

## 5 CONCLUSION

Our study provides ecologists with a modeling approach to infer predatory interactions from observationally collected count data across multiple landscapes. This remarkably flexible approach opens novel opportunities to evaluate species interactions across natural or disturbance gradients, including when species abundances vary or where some sites experienced extirpations. There is direct applicability to ongoing predator conservation and trophic cascades research as our study species continue to experience range contractions. We look forward to opportunities to ground truth the results from our approach by co‐locating camera trapping studies with manipulative experiments (e.g., predator reintroductions) and direct predation observations.

## AUTHOR CONTRIBUTIONS

**Zachary Amir:** Conceptualization (lead); data curation (equal); formal analysis (lead); investigation (lead); methodology (lead); software (lead); validation (lead); visualization (lead); writing – original draft (lead); writing – review and editing (equal). **Adia Sovie:** Formal analysis (supporting); investigation (supporting); methodology (supporting); software (supporting); supervision (supporting); visualization (supporting); writing – review and editing (equal). **Matthew Scott Luskin:** Conceptualization (supporting); data curation (equal); funding acquisition (lead); supervision (lead); writing – review and editing (equal).

## FUNDING INFORMATION

The research was funded by the Smithsonian Institution's ForestGEO program, the Nanyang Technological University in Singapore, the University of Queensland, the National Geographic Society Committee for the Research and Exploration #9384‐13, and numerous small grants for field work. M.S.L. was supported by an Australian Research Council Discovery Early Career Award no. DE210101440.

## CONFLICT OF INTEREST

The authors declare no conflict of interest pertaining to the research conducted here.

### OPEN RESEARCH BADGES

This article has earned Open Data and Open Materials badges. Data and materials are available at https://doi.org/10.5061/dryad.b8gtht7h3.

## Supporting information


Appendix S1

## Data Availability

All R code and data used to implement our Bayesian co‐abundance models can be accessed at this Figshare repository: https://doi.org/10.5061/dryad.b8gtht7h3.
