# Supplementary material for: Inferring predator–prey interactions from camera traps: A Bayesian co‐abundance modeling approach
Source: Ecol Evol. 2022 Dec 12;12(12):e9627. doi: 10.1002/ece3.9627 (PMC9745391; doi:10.1002/ece3.9627)
Supplement: Supplementary file 1 — Appendix S1 [file ECE3-12-e9627-s001.docx]

**Supporting Tables and Figures-**

**Table S1**: Study site description and effort for camera trapping sessions. Trap nights were calculated between the first and last photos taken for each collected camera. MCP refers to the minimum convex polygon around the camera traps in a single session. To account for variation in deployment scale and spacing, we resampled all data by grouping cameras into hexagonal units with a short diagonal of 3 km (7.8 km^2^ cells).

| Survey | Annual rainfall | | Cameras collected | Effort (Trap nights) | Duration | Elevation  (Mean ± SD) | Elevation range | MCP | Camera spacing |
| --- | --- | --- | --- | --- | --- | --- | --- | --- | --- |
| THAILAND |  | |  |  |  |  |  |  |  |
| Khao Chong / Khao Ban Tat 2018 | 2014.28 | | 76 | 3957 | 2018-02-01 – 2018-04-30 | 524.59 ± 270.92 | 103 – 1234 | 59.01 | 467.95 |
| Khao Yai 2019 | 1119.49 | | 61 | 3553 | 2019-07-01 – 2019-09-25 | 769.64 ± 38.56 | 582 – 816 | 22.54 | 464.42 |
| SUMATRA |  | |  |  |  |  |  |  |  |
| Gunung Leuser 2014 | 2828.00 | | 69 | 3401 | 2013-12-18 – 2014-05-22 | 316.03 ± 250.38 | 25 – 888 | 516.15 | 1275.27 |
| Kerinci Seblat 2014 | 2406.94 | | 98 | 5356 | 2014-02-10 – 2014-10-04 | 594.03 ± 194.46 | 252 – 1154 | 813.69 | 1169.04 |
| Bukit Barisan Selatan 2014 | 2987.80 | | 79 | 5750 | 2014-06-15 – 2014-09-20 | 369.75 ± 184.97 | 116 – 935 | 473.58 | 1139.96 |
| MALAYSIAN BORNEO |  | |  |  |  |  |  |  |  |
| Danum Valley 2019  (Sabah) | 2182.68 | | 22 | 1292 | 2019-05-24 – 2019-09-26 | 256.73 ± 102.02 | 184 – 567 | 8.31 | 520.76 |
| Danum Valley 2018  (Sabah) | 2182.85 | | 27 | 1849 | 2018-07-12 – 2018-10-30 | 249.63 ± 53.23 | 175 – 381 | 15.95 | 614.15 |
| Lambir Hills 2017  (Sarawak | 3078.82 | | 67 | 2406 | 2017-05-23 – 2017-07-07 | 164.80 ± 65.31 | 60.31 – 421.44 | 22.06 | 459.95 |
| PENINSULAR MALAYSIA | |  |  |  |  |  |  |  |  |
| Pasoh 2013 | 2081.40 | | 58 | 1399 | 2013-05-29 – 2014-02-12 | 297.09 ± 160.10 | 98 – 674 | 133.53 | 1316.26 |
| Pasoh 2014 | 2079.16 | | 57 | 1314 | 2014-05-13 – 2014-08-01 | 303.14 ± 160.04 | 98 – 674 | 134.62 | 1321.48 |
| Pasoh 2015 | 2079.78 | | 59 | 1670 | 2015-05-07 – 2015-09-04 | 301.15 ± 158.15 | 98 – 674 | 134.62 | 1317.78 |
| Pasoh 2017 | 2086.38 | | 42 | 1305 | 2017-05-17 – 2017-08-29 | 308.98 ± 156.05 | 103 – 674 | 122.63 | 1416.43 |
| Ulu Muda 2015a | 2057.03 | | 76 | 4242 | 2014-11-01 – 2015-01-30 | 278.58 ± 128.68 | 117 – 628 | 68.98 | 938.65 |
| Ulu Muda 2015b | 2063.01 | | 112 | 4446 | 2015-01-31 – 2015-05-01 | 295.77 ± 139.83 | 117-843 | 113.61 | 731.56 |
| Ulu Muda 2015c | 2080.90 | | 52 | 3582 | 2015-05-02 – 2015-07-31 | 325.38 ± 166.15 | 141-843 | 115.53 | 1227.86 |
| Ulu Muda 2015d | 2078.17 | | 48 | 2862 | 2015-08-01 – 2015-10-30 | 328.92 ± 165.27 | 123-843 | 104.01 | 1237.85 |
| Ulu Muda 2016a | 2065.89 | | 73 | 2220 | 2015-10-31 – 2016-01-29 | 313.62 ± 145.27 | 117-748 | 103.17 | 794.92 |
| Ulu Muda 2016b | 2054.55 | | 60 | 2899 | 2016-01-30 – 2016-04-29 | 285.45 ± 135.12 | 117-628 | 66.96 | 958.39 |
| Ulu Muda 2016c | 2060.54 | | 46 | 2746 | 2016-04-30 – 2016-07-22 | 301.30 ± 138.91 | 117-628 | 65.72 | 974.47 |
| SINGAPORE |  | |  |  |  |  |  |  |  |
| Singapore 2019 | 2283.97 | | 28 | 1822 | 2018-12-26 – 2019-03-17 | 41.44 ± 22.18 | 0 – 83 | 162.35 | 261.70 |

**Table S2**: Study species capture information. Captures were considered independent if they occurred greater than 30 minutes from each other. NP stands for national park, CA stands for conservation area, WS stands for wildlife sanctuary, and FR stands for forest reserve. More information about landscapes can be found in the Supporting Information, section 1.

|  | Total number of independent captures | Landscapes Detected |
| --- | --- | --- |
| Tiger  (*Panthera tigris*) | 72 | 1. Gunung Leuser NP 2. Kerinci Seblat NP 3. BBS NP |
| Clouded Leopard (*Neofelis nebulosa* & *N. diardi*) | 113 | 1. Khao Yai NP 2. Ulu Muda Forest 3. Pasoh FR 4. Gunung Leuser NP 5. Kerinci Seblat NP 6. BBS NP 7. Danum Valley CA |
| Muntjac deer  (*Muntiacus muntjak*) | 5358 | 1. Khao Yai NP 2. Ulu Muda Forest 3. Pasoh FR 4. Khao Ban Tat WS 5. Gunung Leuser NP 6. Kerinci Seblat NP 7. BBS NP 8. Danum Valley CA 9. Lambir Hills NP |
| Sambar deer  (*Rusa unicolor*) | 437 | 1. Khao Yai NP 2. Ulu Muda Forest 3. Pasoh FR 4. Singapore 5. Gunung Leuser NP 6. Kerinci Seblat NP 7. BBS NP 8. Danum Valley CA |


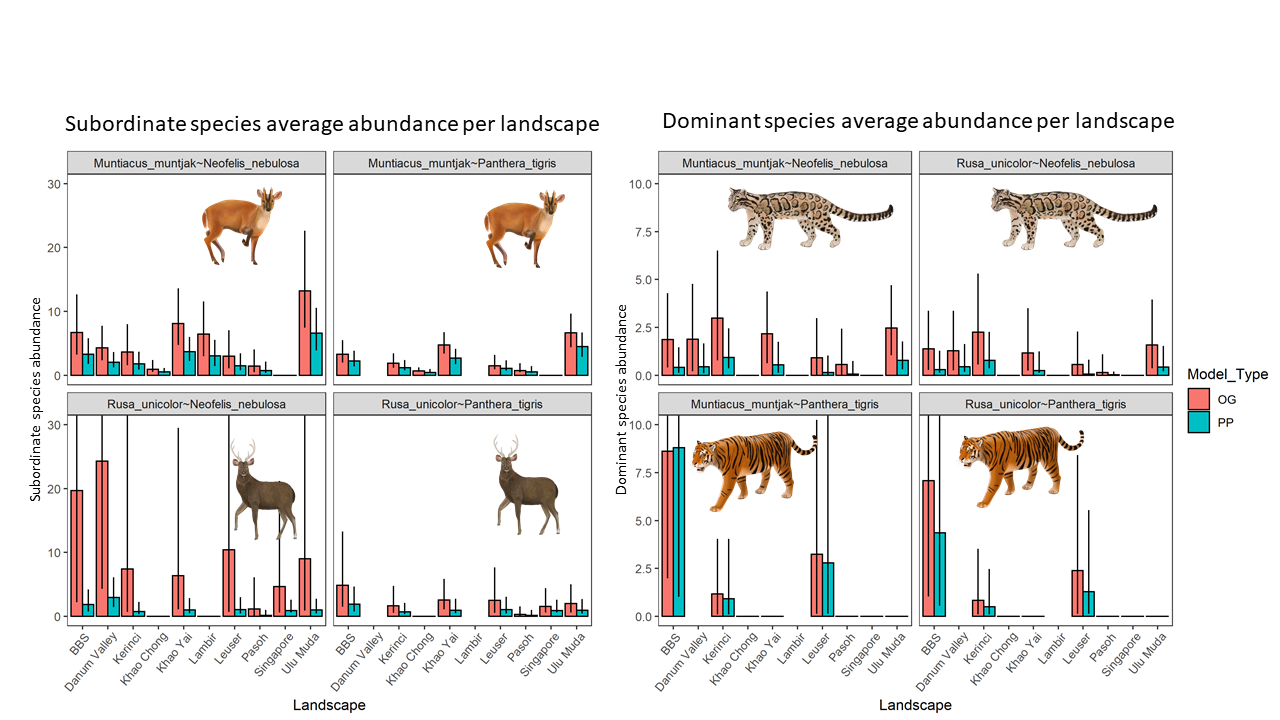


**Figure S1**: Abundance estimates per 7.8 km^2^ sampling unit for both predator and prey averaged across each landscape from co-abundance models that used a Poisson distribution in the detection formula (PP) and from co-abundance models that used a binomial distribution in the detection formula (OG) presented in the main text. All models contain the informed zero-inflated Poisson parameter in the abundance formula and contained the overdispersion random effect in the detection formula. Note the Y-axis for prey species ranges from 0-30, while the Y-axis for predators ranges from 0-10. All prey species and clouded leopards exhibited larger abundance estimates with larger 95% Bayesian credibility intervals around the estimate in the OG co-abundance model. The key exception was for tigers that exhibited similar average abundance estimates per landscape between the PP and the OG co-abundance models. All species showed similar average abundance per landscape trends between both PP and OG co-abundance models.


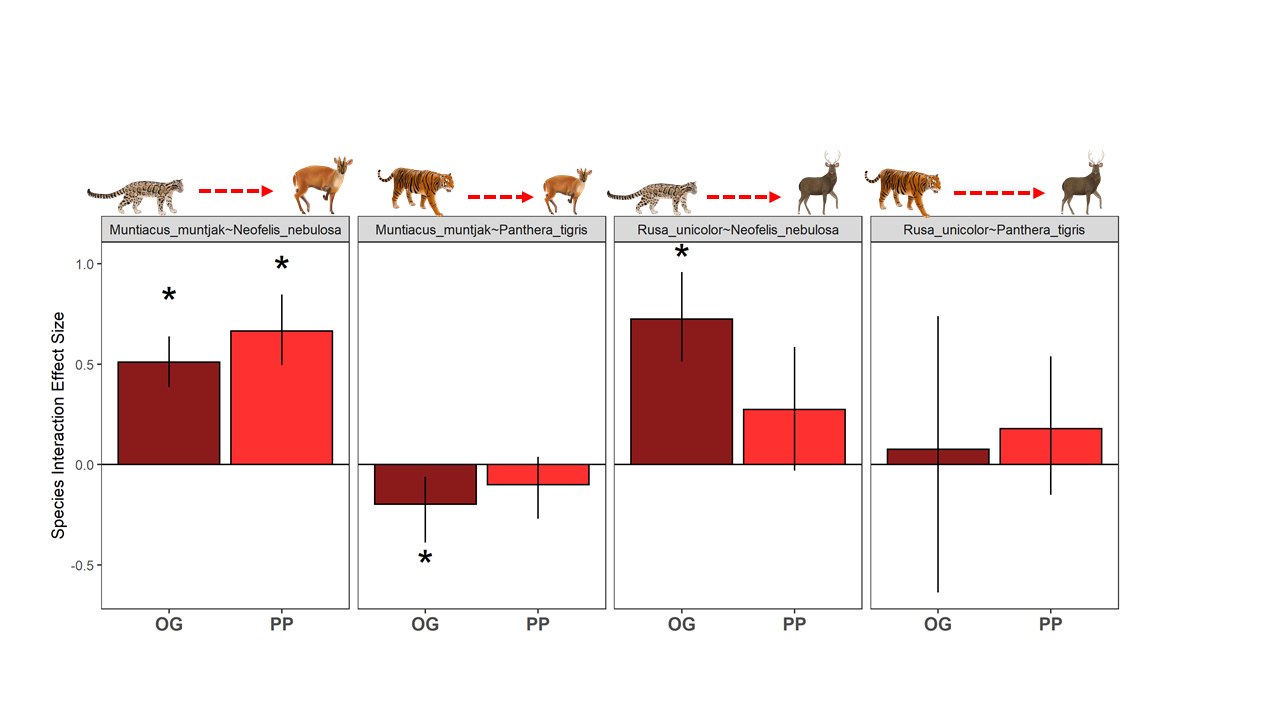


**Figure S2**: The posterior mean effect size of our species interaction parameter from co-abundance models that used a binomial distribution (OG) or Poisson distribution (PP) in the detection formula for both species. The error bars represent the 95% Bayesian credibility intervals (CI) around the mean, asterisks (*) denote clear relationships (i.e., 95% CI does not include zero), and all four species pairs are represented. Both OG and PP co-abundance models contain the informed zero-inflated Poisson parameter in the abundance formula and contained the overdispersion random effect in the detection formula. Species interaction parameters showed the same directionality regardless of approach used, though the effect sizes were typically diminished and had larger CIs in the PP co-abundance models.


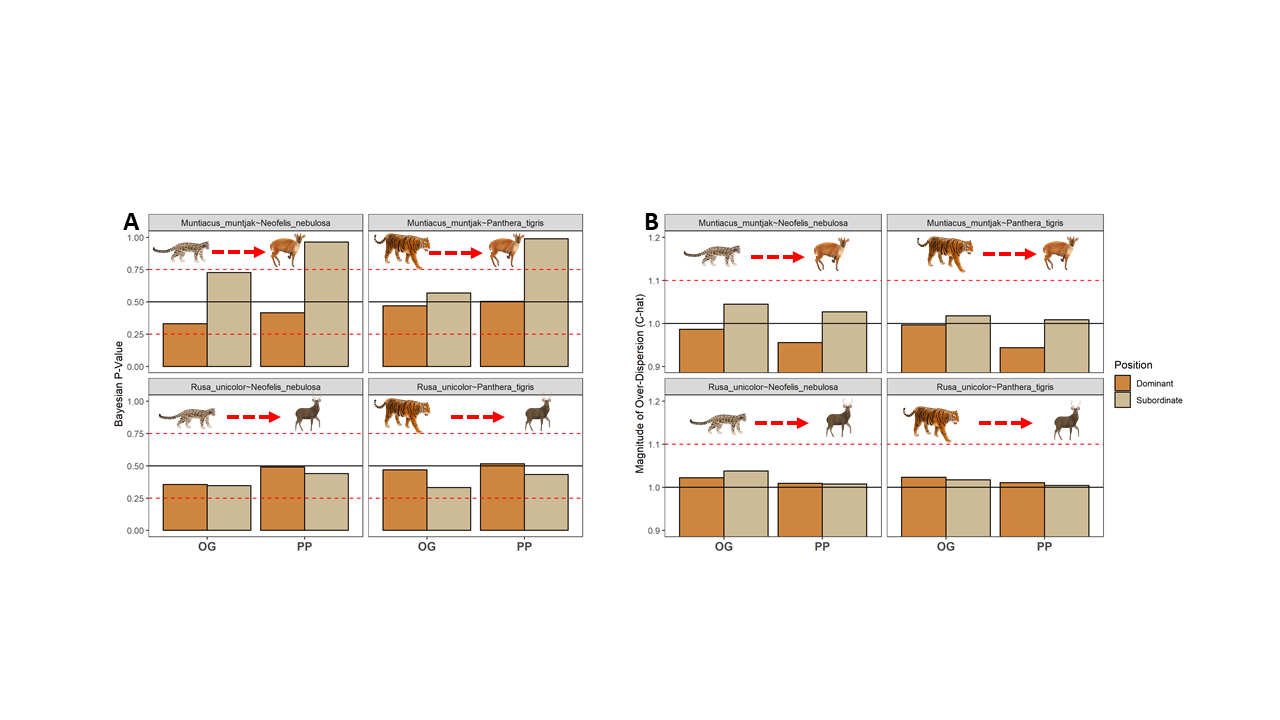


**Figure S3**: Comparing measures of goodness-of-fit between co-abundance models using a binomial distribution in the detection formula (OG) and co-abundance models using a Poisson distribution in the detection formula (PP). **A**) The Y-axis represents Bayesian P-values, which were calculated by taking the mean value of number of times data simulated from the joint posterior distribution was greater than the real data supplied to the model, where Bayesian p-values between 0.25 – 0.75 indicate good fit, a value of 0.5 indicates a perfect fit, and values above or below the dashed red lines (<0.25 or >0.75) indicate a lack of fit. Bayesian P-values between OG and PP co-abundance models indicate that all OG co-abundance models produced good model fit for both dominant and subordinate species, while the PP co-abundance model produced poor model fit for when muntjac deer was subordinate. **B**) The Y-axis represents our C-hat values, which we calculated by dividing the observed data supplied to the model from data simulated from the joint posterior distribution and took the mean value, where c-hat values that are greater than 1.1 indicate remaining overdispersion and values close to 1 indicate no remaining overdispersion. A horizontal line is added at 1 to indicate the ideal value for our C-hat scores, while the red dashed line at 1.1 denotes our cut-off point for C-hat values that suggest overdispersion. Both OG and PP co-abundance models showed no remaining overdispersion for either dominant or subordinate species.


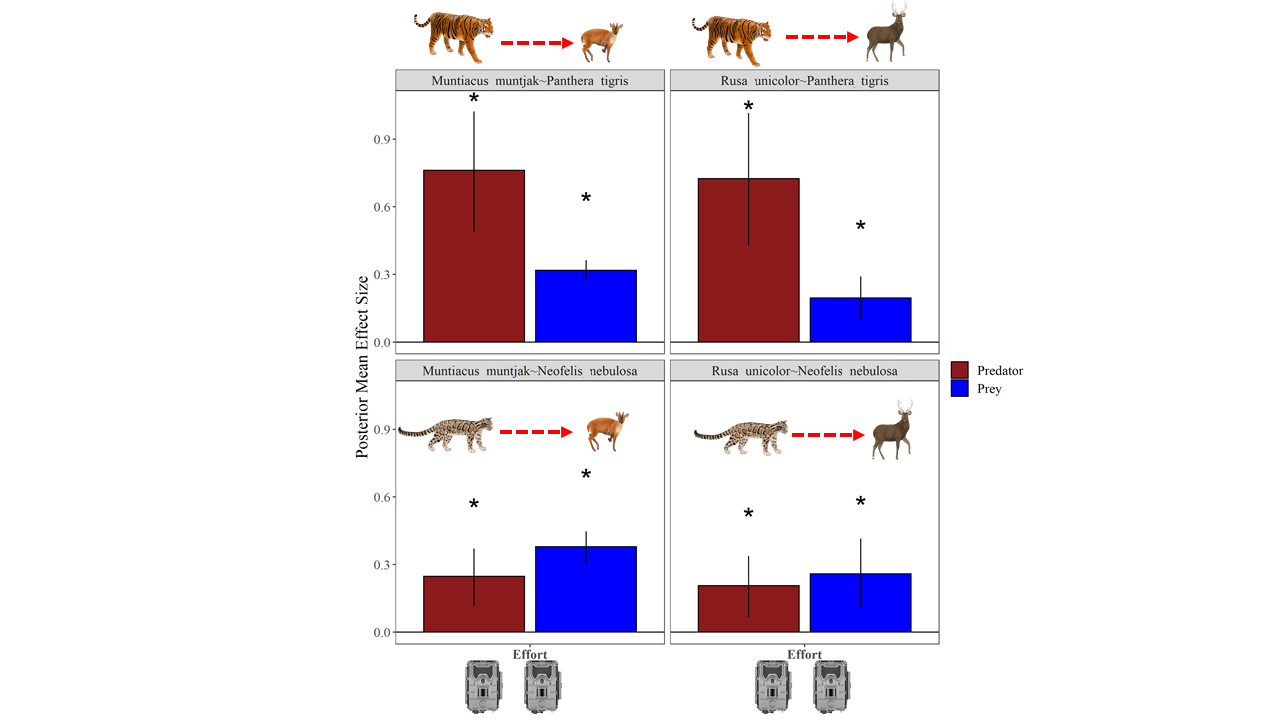


**Figure S4**: Bar plots depicting the posterior mean effect size of the effort parameter on both predator and prey detection probability in the co-abundance model that included the informed zero-inflated Poisson distribution and the overdispersion random effect. All species across all models showed a clear positive response with increased effort per sampling unit. Error bars show the 95% Bayesian credibility interval (CI), and asterisks (*) denote clear relationships (i.e. 95% CI does not include zero). **A**) describes the model of tiger abundance impacting muntjac deer abundance, **B**) is tiger abundance impacting sambar deer abundance, **C**) is clouded leopard abundance impacting muntjac deer abundance, and **D**) is clouded leopard abundance impacting sambar deer abundance.
